# The Hospital Movement in the United States

**Published:** 1905-12-09

**Authors:** 


### HOSPITAL ADMINISTRATION.

### CONSTRUCTION AND ECONOMICS.

### THE HOSPITAL MOVEMENT IN THE UNITED STATES.

### THE MASSACHUSETTS STATE HOSPITAL AT TEWKSBURY.

By our Special Commissioner.

### I. DESCRIPTIVE AND EXPLANATORY.

It may be well to explain that the word "Hospital" may be used in the United States indiscriminately to denominate institutions for (1) the cure of disease; (2) the treatment of the insane, and (3) the almshouse or workhouse for the reception of paupers of all types and conditions.

The almshouse portion of the State Hospital at Tewksbury, Mass., provides accommodation for the aged and thriftless poor—mostly from the old and from foreign countries be it said, to the credit of the American people—their children, accidents, and surgical and medical cases of the pauper class; maternity cases, mostly unmarried women; and insane patients. The "workhouse" means in America the penitentiary, which is a prison where insane criminals, tramps and drunks are received. Thus, in Massachusetts, people are sentenced to the State Farm or Workhouse at Bridgewater. We think it well to make this point clear.

The buildings of the State Hospital at Tewksbury stand upon an excellent site, parts of which are beautifully timbered, and there is a large farm in connection with the establishment cultivated by pauper labour, which supplies the establishment with milk, eggs, beef, corn, fruit and vegetables.

#### Plan of the State Hospital.

Immediately to the right and in front of the hospital is the Medical Superintendent's house. The main entrance is through the administration block and to the right of it is placed the almshouse for women, a new and well planned building of modern type; behind it is the maternity department; and further back is situated the hospital for females in two blocks connected by a wide corridor which constitutes an airy, well-lighted day room for convalescents. Further to the right and behind the maternity block is a large laundry fitted with modern machinery worked by electricity. On the immediate left of the administration block is situated the hospital chapel. Further to the left there is the almshouse for men, with the hospital for male patients, and lastly a modern, well-equipped operation pavilion, a mortuary, and other buildings. The space between the male and female departments is laid out as a garden, which contains open pavilions for the use of the patients in fine weather and is most spacious and attractive in appearance. At the back of this garden space are situated the insane wards for men and women, the former having been built and opened for patients on January 1, 1904, and the latter more recently. This department of the establishment constitutes a modern insane hospital where the day rooms, especially, and their appurtenances are attractive and good. Indeed, the whole arrangements reflect the greatest credit upon Dr. Nichols, the Superintendent and resident physician.

Speaking of the buildings as buildings, we should be justified in defining them (taken as a whole) as being equal to anything of the kind in the world. This fact proves the deep impression which was made by the crusade levelled against the administration of this very institution, some twenty years ago, by the then Governor of Massachusetts, the late General Butler. We understand that General Butler won his election as Governor by his crusade against the scandals and abuses which characterised the management of the State Hospital in his day. The only monument, in our opinion, which is worthy of a great man is the continuous vitality of the principles which he strove to enforce during his lifetime. Holding these views, we think the people of Massachusetts, as well as the late General Butler's family and descendants, may well rest content with the high efficiency of the State Hospital at Tewksbury, for no man could wish for a more striking monument to his public work, than the late General Butler has in the present condition of this Institution.

#### Americans and Paupers.

The American people have the strongest contempt for any man or woman who gravitates to pauperism. From our observation we should say, that they are justified in this feeling, from the fact, that no person with any intelligence, who is industrious and possessed of character, ought to fail to provide the necessaries of life, in reasonable proportion, in the United States to-day, without any extraordinary output of energy or personal wear and tear. The opportunities for improving one's position, offered by the United States, are probably greater than those presented by any other country in the world. There is, too, a wholesome family life, so far as the vast majority of the population is concerned, which insures that any sick or dependent member shall be cared for by the family. We mention these facts because they reflect great credit upon the public spirit which provides such excellent accommodation for the paupers found within the State of Massachusetts, seeing that only 898 inmates, out of a total of 3,901 during the last year (1904) for which the figures are available, were born in Massachusetts. Of the total, 2,406, or upwards of three-fifths, were foreigners, of which nearly one-half were born in Ireland, 195 in England, the remainder being natives of Italy, Russia, Scotland,

Sweden, Germany, Poland, Finland, Austria, Armenia, Turkey, Greece, France, Denmark, Norway, Portugal, Cuba, Roumania, Belgium, Wales, Mexico, Spain, Servia, Australia, Guernsey, Holland, Egypt, and even China. This list coupled with the figures constitutes an object lesson of the cost entailed upon a popular nation which is being rapidly developed by emigrants from all parts of the world. We visited the institution on October 23, 1905, when there were 1,284 inmates of whom only 320 were inmates of the almshouse, that is, pauper residents, and of these a considerable number were convalescents discharged from the hospital proper. Of the remainder, 69 were children who are educated in a special school building apart from the hospital; 472 were insane, 293 were undergoing treatment for disease, of which 184 were men and 109 women, and 103 patients were suffering from tuberculosis, and were under treatment in a special sanatorium.

### Department for the Insane.

Knowing that the State of Massachusetts contains nearly 10,000 people who are under treatment for insanity, of whom nine-tenths are provided for in hospitals for the insane—of which there are twelve in the State—we were curious to know why this class of case should be received at the almshouse. Of the insane patients about one-third are chronics sent from insane hospitals within the State, and about two-thirds are patients declared insane after admission. It appears then that the majority of the cases are first sent to the almshouse, where they are placed under medical care, and are subsequently certified to be insane. The almshouse is the natural refuge of all destitute persons who have no visible means of subsistence, and every case sent in from any part of the State, which is not chargeable to any city or township possessing an almshouse, is admitted as a matter of course. There are under treatment in the insane department a number of acute cases of various types, but the majority of the patients are chronic dements whose labour is of appreciable value on the farm, in the laundry, and in various departments of the establishment. We take it that the average American is a smart man of business, and no doubt the actual money value of the labour rendered by the inmates of the insane department tends materially to reduce the cost per head of each inmate. In any case, the accommodation provided for the insane is equal to that of the best English county asylums, and so may be regarded as of the best.

*(To be continued.)*

